# The Role of Stress Modifier Biostimulants on Adaptive Strategy of Oregano Plant for Increasing Productivity under Water Shortage

**DOI:** 10.3390/plants12244117

**Published:** 2023-12-09

**Authors:** Reza Abdali, Amir Rahimi, Sina Siavash Moghaddam, Saeid Heydarzadeh, Carmen Arena, Ermenegilda Vitale, Mohammad Zamanian

**Affiliations:** 1 Department of Plant Production and Genetics, Faculty of Agriculture and Natural Resources, Urmia University, Urmia P.O. Box 165-57153, Iran; r.abdali@urmia.ac.ir (R.A.); a.rahimi@urmia.ac.ir (A.R.); s.moghadam@urmia.ac.ir (S.S.M.); 2 Department of Plant Production and Genetics, Faculty of Agriculture and Natural Resources, University of Mohaghegh Ardabili, Ardabil P.O. Box 179, Iran; 3 Department of Biology, University of Naples Federico II, 80126 Napoli, Italy; ermenegilda.vitale@unina.it; 4 NBFC—National Biodiversity Future Center, 90133 Palermo, Italy; 5 Seed and Plant Improvement Institute, Agricultural Research, Education and Extension Organization (AREEO), Karaj P.O. Box 31585-4114, Iran; m.zamanian@areeo.ac.ir

**Keywords:** sustainable agriculture, antioxidant, optimal irrigation, chitosan, seaweed

## Abstract

To investigate the influence of stress modulators on the adaptive physiological responses and biomass traits of oregano under water stress conditions, a two-year (2018 and 2019) randomized complete block-designed factorial research was performed. In this study, oregano plants were treated with five stress modulators levels (CHN: chitosan, AMA: amino acids, SEW: seaweed, ASA: ascorbic acid, SAA: salicylic acid, and CON: control) at three levels of irrigation regimes (Irr40 (40), Irr60 (60) and Irr75 (75) % field capacity). The effects of water shortage and biostimulant application were evaluated on total dry weight (TDW), relative water content (RWC), essential oil production, chlorophyll, nutrient (N, K, and P), proline, total soluble sugar, polyphenol and flavonoid content, and activity of antioxidant enzymes. The result showed that under optimal irrigation conditions, oregano plants sprayed with CHN exhibited the highest dry weight (141.23 g m^−2^) as a morphological trait, the highest relative water content (79.34%), the most consistent concentrations of nitrogen, phosphorus and potassium (3.14, 0.39, and 1.69%, respectively), chlorophylls a and b (3.02 and 1.95 mg g^−1^ FW, respectively), and total phenols and total flavonoids (30.72 and 3.17 mg g^−1^ DW, respectively). The water deficit increased the proline content, with the greatest amount (4.17 mg g^−1^ FW) observed in control plants. Moreover, under moisture shortage stress conditions, the application of CHN and SEW increased the soluble sugar (27.26 mg g^−1^ FW) and essential oil yield (1.80%) production, the catalase, ascorbate peroxidase, and superoxide dismutase activities (3.17, 1.18, and 63.89 μmol min^−1^ g^−1^ FW, respectively) compared to control plants. In summary, the study demonstrated that oregano plants respond positively to stress modulator treatments when subjected to moisture shortage stress, especially when treated with chitosan. The results offer promising insights for developing sustainable adaptative strategies aimed at enhancing the oregano’s tolerance to water shortage, ultimately improving its productivity and biochemical traits.

## 1. Introduction

Oregano (*O. vulgare* L.) is a fragrant and perennial plant belonging to the Lamiaceae family that originates from the Mediterranean region and has numerous applications in the food and pharmaceutical industries. In addition to its wide use as a spice and condiment, oregano is known for the unique composition of its valuable essential oil, primarily found in the inflorescences, leaves, and stems of plants [1]. The aerial parts of the plant are significant sources of polyphenols, flavonoids, triterpenoids, carvacrol, and thymol, compounds known for their strong bioactivity [2]. As a result of the presence of numerous secondary metabolites, oregano offers antifungal, antibacterial, antiviral, and anticancer properties [3]. This plant can easily withstand drought stress, but it should be watered regularly and adequately to increase the yield [2,4].

On a global scale, the ongoing climate changes are evident in the increasing annual maximum temperatures and rising aridity. The insufficient availability of water, both in terms of quantity and its distribution during plant growth, leads to a diminished realization of the plant’s full genetic potential. This ultimately reduces the yield and income for the farmer [3]. Moisture shortage stress is a key limiting abiotic factor that affects nearly all aspects of the morphology, physiology, plant development, growth, and productivity in arid and semi-arid regions [5]. Moisture deficit stress induces metabolic and physiological reactions such as reduction in plant development and growth, stomatal conductance, dry matter accumulation, chlorophyll content, growth rate, leaf water potential, and assimilation [6]. Under water deficit stress, plants usually respond by osmotic adjustment, stomata regulation, and antioxidant defense to reduce stress-related damages [7]. The osmotic adjustment is defined as a procedure of solute accumulation in dividing cells when the water potential is decreased, thereby preserving the cells’ turgor. This is considered a kind of adaptation to water scarcity to diminish the damage derived from moisture deficit stress [3,5]. Various research has described the increased reactive oxygen species (ROS), particularly under moisture shortage stress [6,8]. Plants mitigate ROS production through both enzymatic and non-enzymatic antioxidant mechanisms [9]. Once accumulated within cells, ROS can damage proteins, membrane lipids, and nucleic acids, compelling the activation of enzymatic and non-enzymatic antioxidant systems to counter ROS-induced oxidative stress [10]. Considering the anticipated significant reduction in water resources in the future, water conservation becomes vital in oregano farming [5]. Therefore, innovative equipment aimed at reducing moisture deficit stress and enhancing tolerance is currently under investigation [11].

Agricultural systems have increasingly concentrated on sustainable, organic, and ecologic crop production. Among the approaches applied, there are plant biostimulants. Biostimulants are products involved in the stimulation of plant nutritional processes [7,11]. The objective of their application is to advance the functioning of the plant, e.g., abiotic stress tolerance, nutrient efficiency, and humification. The biostimulants trigger the reduction of fertilizers while enhancing the plant’s tolerance to biotic and abiotic pressures [11]. Biostimulants are mainly derived from various origins, such as diverse organic materials and their combinations [12,13]. Seaweed extract, which is generally employed for different plants, is crucial since it comprises a significant quantity of vitamins, organic substances, fatty acids, and microelements, besides existing rich in plant growth regulators like gibberellins, auxins, and cytokinin [11]. Seaweed has auspicious effects, in terms of improving tolerance to environmental stress, enhancing plant growth and development, and increasing antioxidant personalities in plants [7]. Amino acids used for plants are mainly derived from enzymatic catalysis [11]. Free amino acids with a small molecular weight are particularly significant since they are rapidly absorbed by crops [12] and act as organic carriers and chelators, facilitating highly efficient nutrient supply to plants [14]. They form identical electrically apathetic and tiny particles with nutrients, crucial for enhancing the uptake and transport of nutrients throughout the plants’ system, especially under conditions of moisture deficit pressure [14].

Ascorbate, commonly known as vitamin C, is not only involved in the ascorbate–glutathione pathway but also protects enzymes with prosthetic transition metal ions [15]. It acts as a cofactor for various enzymes, including those implicated in cell wall division and the hydroxylation of proline residues [16]. Furthermore, the accumulation of H_2_O_2_ can trigger the activation of alternative oxidase, which may provide resources for safeguarding cells against oxidation [9].

The application of salicylic acid (SAA) at various concentrations through seed soaking, root administration or foliar spraying, has been found to alleviate the negative effects of water deficit on stomatal conductance, tissue water status, plant physiological processes, membrane properties, and chlorophyll content [17]. Exogenously applied SAA boosts the activity of antioxidant enzymes and the production of antioxidant compounds, increases plant resistance to abiotic stresses [13], and reduces the lipid peroxidation of the cell tissues and membranes [18].

As far as we know, there is no documented knowledge on the effects of stress modifiers like CHN, AMA, SEW, ASA, and SAA on *O. vulgare* L. when subjected to moisture shortage stress. Therefore, this study aims to assess the influences of stress modifiers such as CHN, AMA, SEW, ASA, and SAA on the adaptive physiological responses and biomass characteristics of *O. vulgare* L. when exposed to water stress.

## 2. Results

### 2.1. Total Dry Weight (TDW), Essential Oil Yield (EOY), Relative Water Content (RWC), Chlorophyll a and b (Chl a and Chl b) Content

The combined analysis of 2 years’ data for the TDW and RWC of *O. vulgare* L. was influenced by the simple effects of the year, irrigation regime, and stress modifier treatments. The interaction influence of ‘irrigation regime × stress modifier’ and year was significant at the 1% probability level (Table 1). The EOY and the content of Chl a and Chl b were influenced by the irrigation regime and stress modifier treatments, and their interaction at 1% levels of probability (Table 1).

#### 2.1.1. TDW

**Figure 1:**
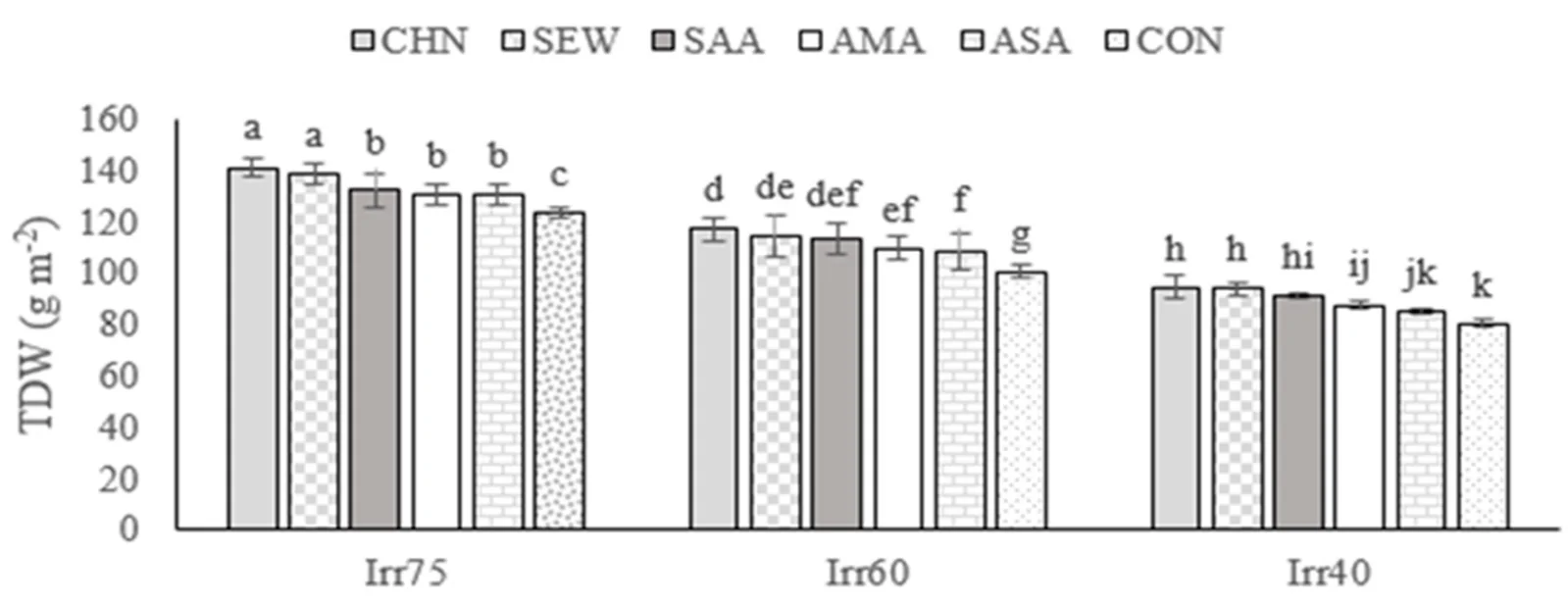
Means comparison and the interactive influence of stress modulators and irrigation regime application on TDW. Similar letters indicate non-significant difference by LSD test at *p* ≤ 0.05. CHN, chitosan; AMA, amino acids; SEW, seaweed; ASA, ascorbic acid; SAA, salicylic acid; CON, control; Irr40, 40, Irr60, 60, and Irr75, 75% field capacity.

The average TDW in the first-year experiment was 109.35 g m^−2^, while in the second year, it was significantly increased, up to 112.08 g m^−2^ (Table 1). It decreased with increasing shortage irrigation regimes (Table 1) and was improved by the application of all stress modulators in comparison with the control plants (Table 1). In particular, the highest TDW (141.23 g m^−2^) was obtained for plants treated with CHN and receiving optimal irrigation, not differing significantly from SEW treatment. The lowest TDW (80.2 g m^−2^) was recorded in the control plants under severe deficit irrigation conditions (Figure 1).

#### 2.1.2. EOY

**Figure 2:**
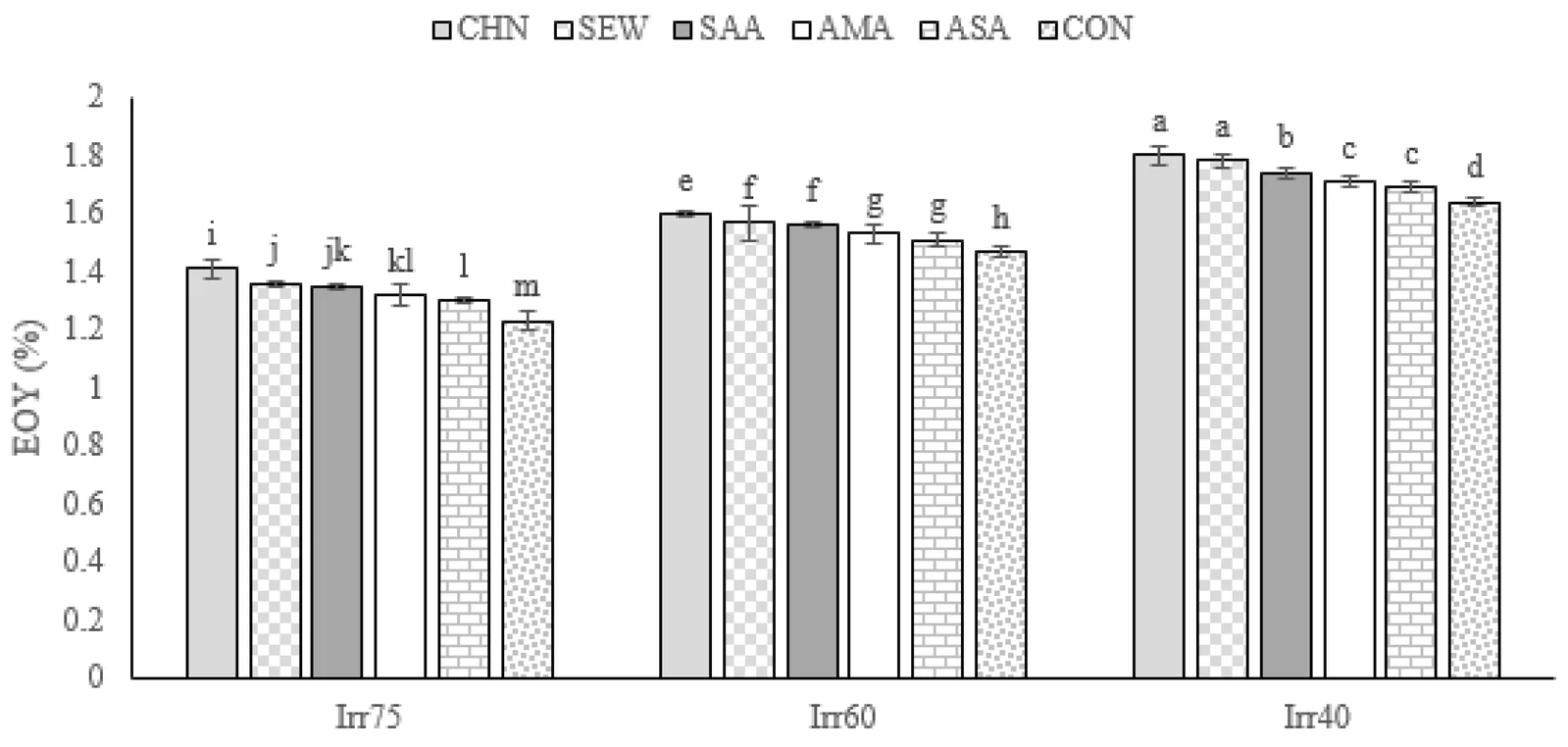
Means comparison and the interactive influence of stress modulators and irrigation regime use on EOY of oregano. Similar letters indicate non-significant difference by LSD test at *p* ≤ 0.05. CHN, chitosan; AMA, amino acids; SEW, seaweed; ASA, ascorbic acid; SAA, salicylic acid; CON, control; Irr40, 40, Irr60, 60, and Irr75, 75% field capacity.

The EOY was higher under Irr40 than Irr75 and Irr60 (Table 1). All biostimulants improved the EOY irrespective of the year and irrigation regime and the CHN induced the most beneficial effect (Table 1). The interaction Irr × Str significantly affected EOY (Table 1). More specifically, the highest EOY of 1.80% was observed in plants sprayed with CHN under the Irr40 regime. The lowest EOY (1.23%) was achieved in the control under optimal irrigation conditions (Figure 2).

#### 2.1.3. RWC

**Figure 3:**
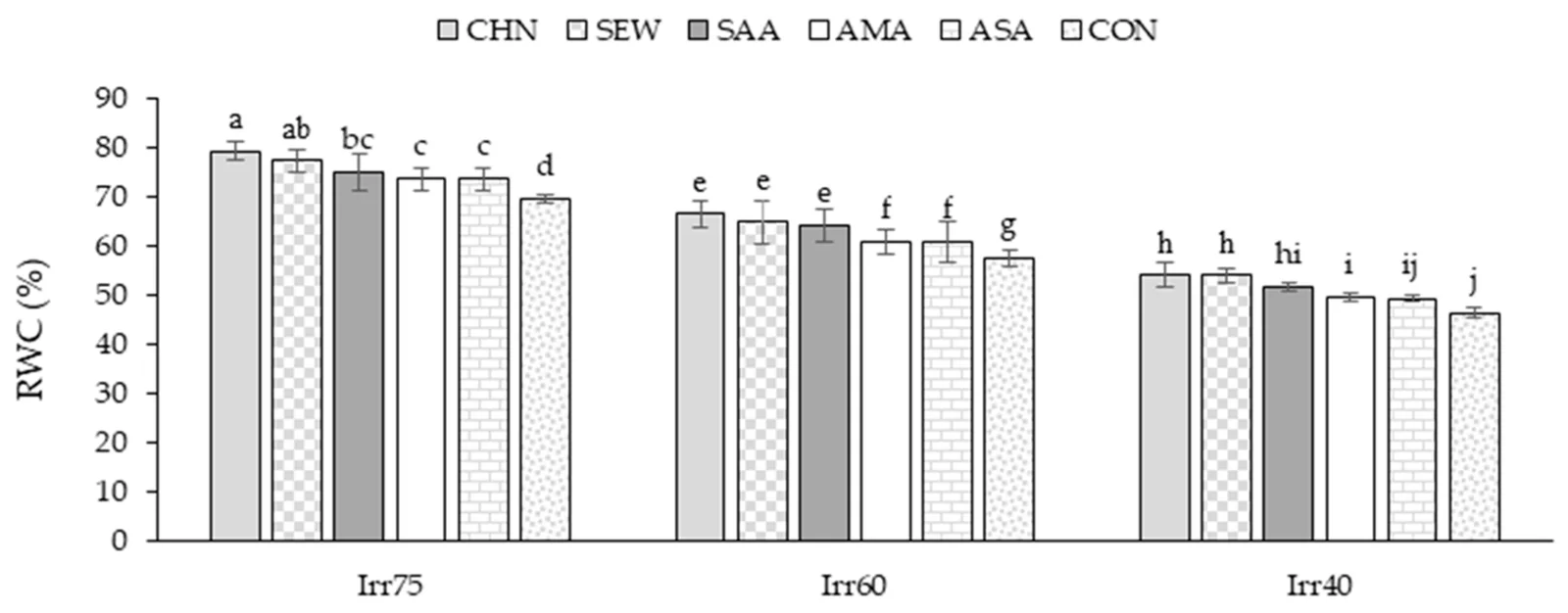
Means comparison and the interactive influence of stress modulators and irrigation regime application on RWC. Similar letters show non-significant difference by LSD test at *p* ≤ 0.05. CHN, chitosan; AMA, amino acids; SEW, seaweed; ASA, ascorbic acid; SAA, salicylic acid; CON, control; Irr40, 40, Irr60, 6, and Irr75, 75% field capacity.

The average RWC in the experiment of the first year was 61.92%, while in the second year, it was 63.48% (Table 1). The RWC showed higher values under optimal irrigation conditions (Table 1). Concerning the stress modulators, the RWC was significantly promoted by CHN (Table 1). Under optimal irrigation, the RWC showed higher values than in moisture shortage stress regimes (Figure 3). The highest RWC of 79.34% was achieved in plants under optimal irrigation and treated with CHN. The lowest (46.44%) was achieved in control plants under Irr40 (Figure 3).

#### 2.1.4. Chl a and Chl b

**Figure 4:**
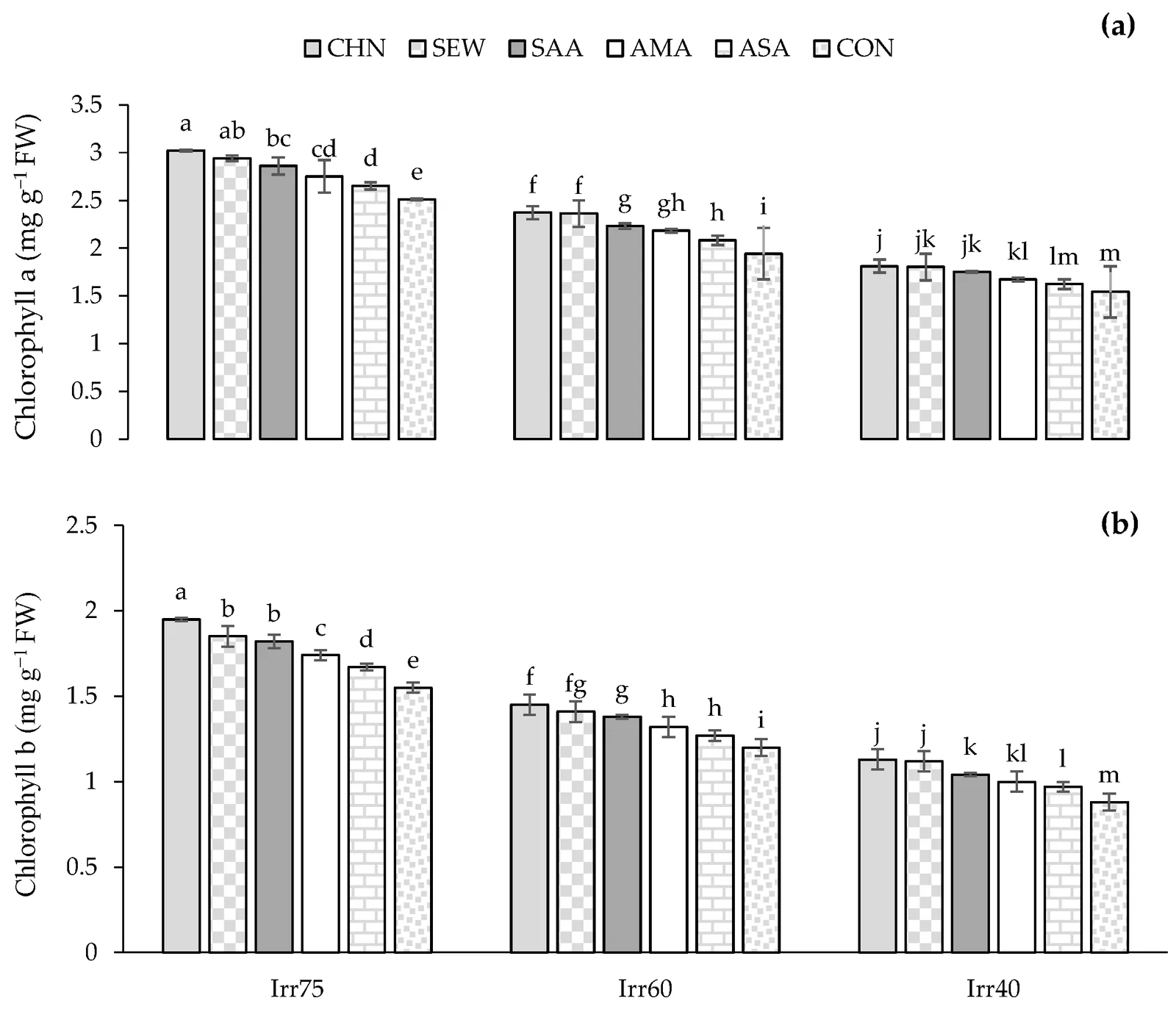
Means comparison and the interactive influence of stress modulators and irrigation regime use on the content of Chl a (**a**) and b (**b**). Similar letters indicate non-significant difference by LSD test at *p* ≤ 0.05. CHN, chitosan; AMA, amino acids; SEW, seaweed; ASA, ascorbic acid; SAA, salicylic acid; CON, control; Irr40, 40, Irr60, 60, and Irr75, 75% field capacity.

The optimal irrigation regime resulted in increased levels of Chl a and Chl b regardless of the type of stress modulator (Table 1). Among biostimulants, CHN led to the highest Chl a and Chl b concentrations (Table 1). In optimal irrigation conditions, Chl a and Chl b amounts were higher than in limited irrigation regimes (Figure 4a,b). The maximum concentrations of Chl a and Chl b (3.02 and 1.95 mg g^−1^ FW) were observed in plants sprayed with CHN in Irr75, whereas the minimum values (1.54 and 0.88 mg g^−1^ FW, respectively) were found in control plants under Irr40 (Figure 4a,b).

### 2.2. Total Soluble Sugar (TSS) and Proline Content (Pro), Antioxidant Enzymes Activity, Total Phenols (TPC), Flavonoids (TFC) Content, Nutrients of N, K, and P

The combined analysis of 2 years’ data for the Pro, CAT, APX, SOD activity, TPC, and TFC of *O. vulgare* L. was influenced by the simple effects of the year, irrigation regime, and stress modifier treatments, and by the interaction ‘irrigation regime × stress modifier’ at 1% levels of probability (Table 2). According to the combined ANOVA of 2 years, the simple effects of the irrigation regime, and stress modifier treatments and their interaction had a notable impact on the TSS, N, P, and K at 1% levels of probability (Table 2).

#### 2.2.1. TSS and Pro Content

**Figure 5:**
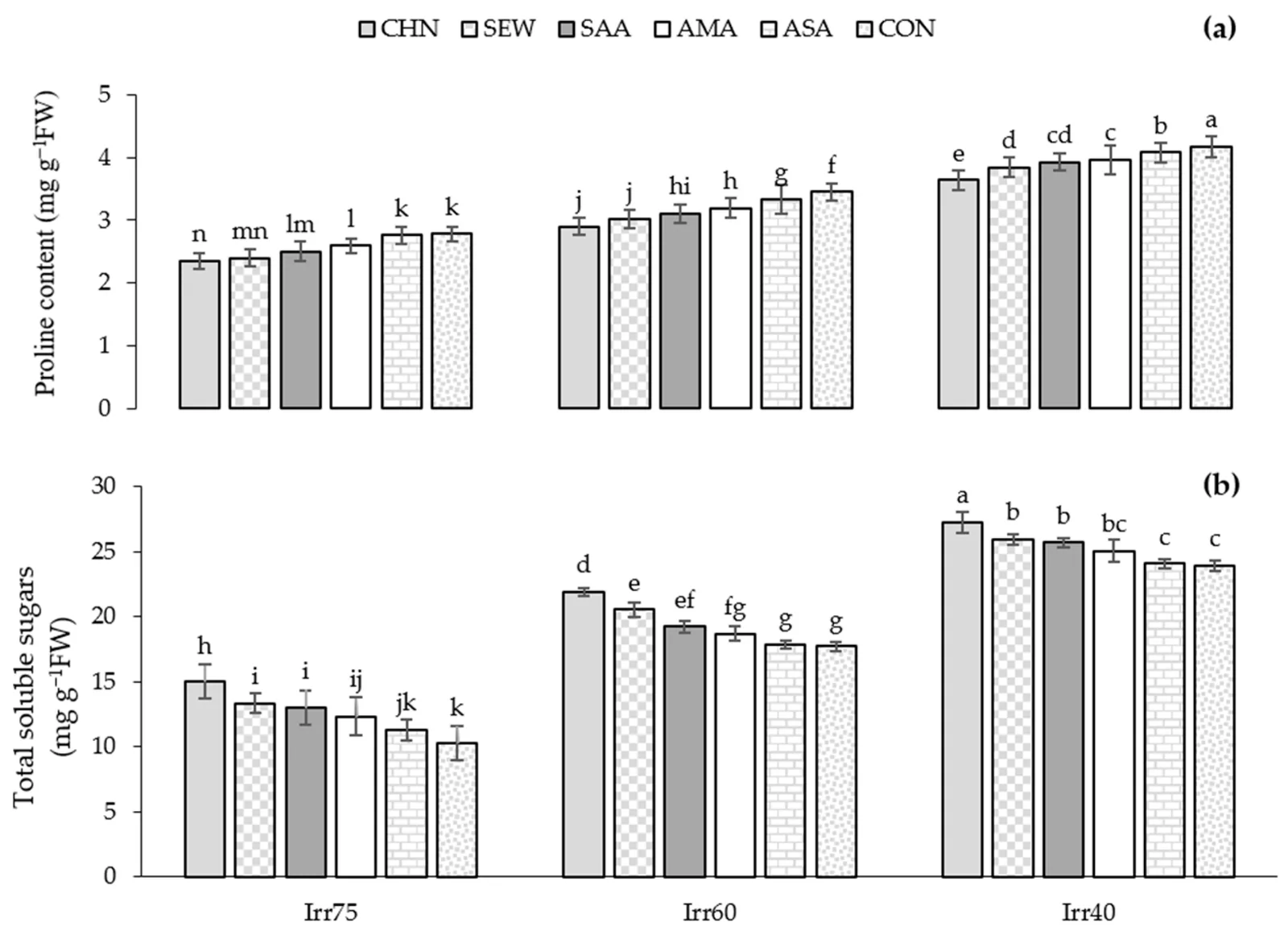
Means comparison and the interactive influence of stress modulators and irrigation regime use on the content of Pro (**a**) and TSS (**b**). Similar letters indicate non-significant differences by LSD test at *p* ≤ 0.05. CHN, chitosan; AMA, amino acids; SEW, seaweed; ASA, ascorbic acid; SAA, salicylic acid; CON, control; Irr40, 40, Irr60, 60, and Irr75, 75% field capacity.

The concentration of leaf Pro was significantly higher in 2018 than in 2019 (Table 2). According to the means comparison, the content of Pro in Irr40 was greater than in Irr75 and Irr60 (Table 2). Thus, the application of stress modulators resulted in a decrease in pro-line content (Table 2). The highest proline content (4.17 mg g^−1^ FW) was achieved in control plants under Irr40, while the lowest (2.35 mg g^−1^ FW) in plants receiving CHN and subjected to the Irr75 regime (Figure 5a). The concentration of TSS in Irr40 was greater than in Irr75 and Irr60 (Table 2). Among the stress modulators, CHN determined the highest TSS (Table 2). The concentrations of TSS in severe scarcity irrigation were higher than under optimal irrigation (Figure 5b). In particular, the greatest concentration of TSS (27.26 mg g^−1^ FW) was recorded in plants treated with CHN under the Irr40 regime and the lowest (10.28 mg g^−1^ FW) in control plants receiving Irr75 (Figure 5b).

#### 2.2.2. CAT, SOD, and APX Activities

**Figure 6:**
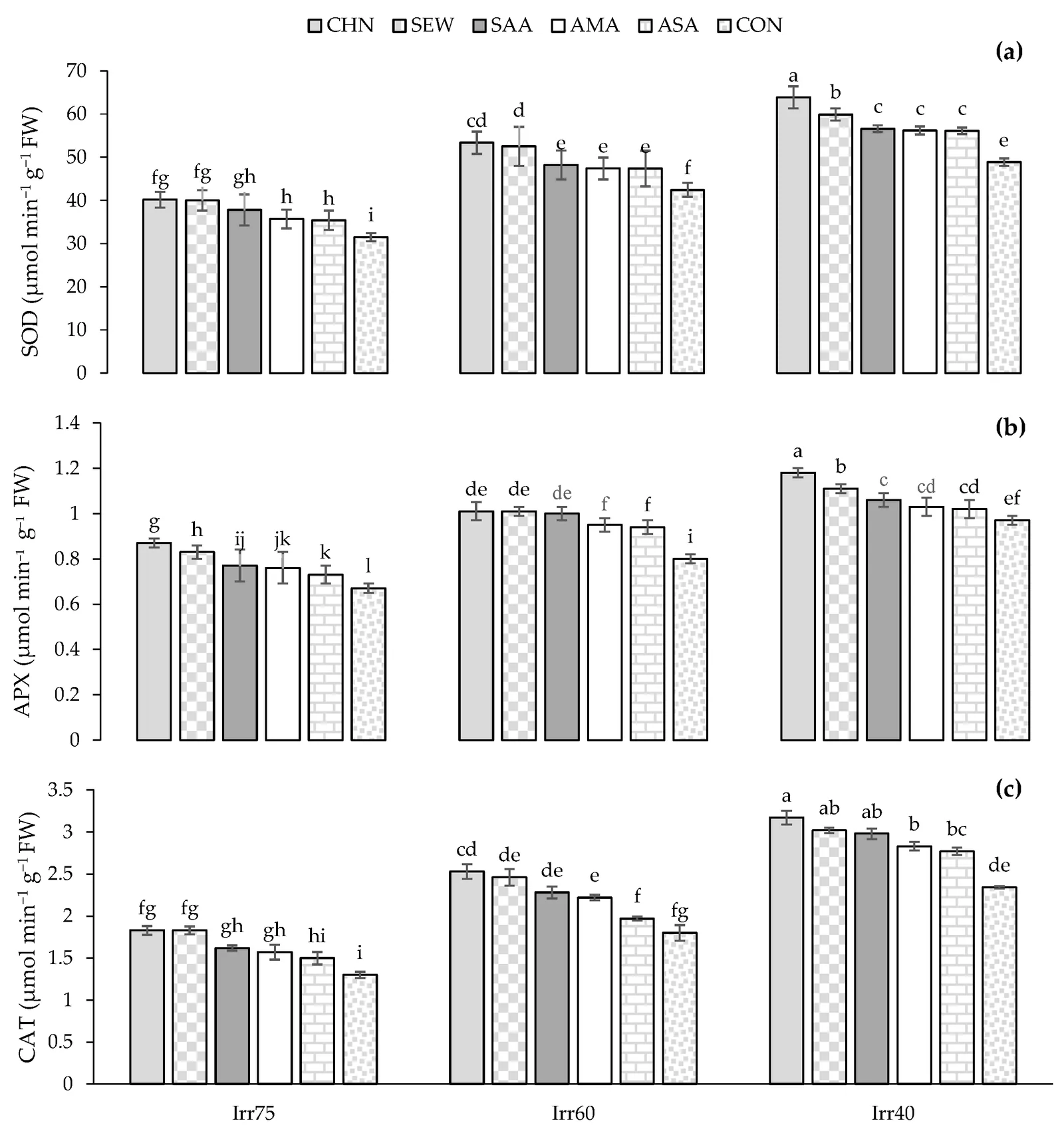
Means comparison and the interactive influence of stress modulators and irrigation regime use on the activity of CAT, catalase (**a**); SOD, superoxide dismutase (**b**); APX, ascorbate peroxidase (**c**). Similar letters indicate non-significant difference by LSD test at *p* ≤ 0.05. CHN, chitosan; AMA, amino acids; SEW, seaweed; ASA, ascorbic acid; SAA, salicylic acid; CON, control; Irr40, 40, Irr60, 60, and Irr75, 75% field capacity.

The average activities of CAT, SOD, and APX of oregano plants were 2.37, 48.17, and 0.95 μmol min^−1^ g^−1^ FW in 2018 and 2.20, 46.66, and 0.91 μmol min^−1^ g^−1^ FW in 2019, respectively (Table 2). The activities of CAT, APX, and SOD under Irr40 were higher than in Irr60 and Irr75 (Table 2). Concerning the stress modulators, the enzyme activities were significantly promoted by CHN and SEW (Table 2). The highest activities of CAT, APX, and SOD (3.17, 1.18, and 63.89 μmol min^−1^ g^−1^ FW) were observed in plants receiving CHN under the Irr40 regime, whereas the lowest activities (1.30, 0.67, and 31.49 μmol min^−1^ g^−1^ FW) emerged from control plants under Irr75 (Figure 6a–c). However, the activities of CAT, APX, and SOD in limited irrigation plants were stronger than in plants under optimal irrigation (Figure 6a–c).

#### 2.2.3. TPC and TFC

**Figure 7:**
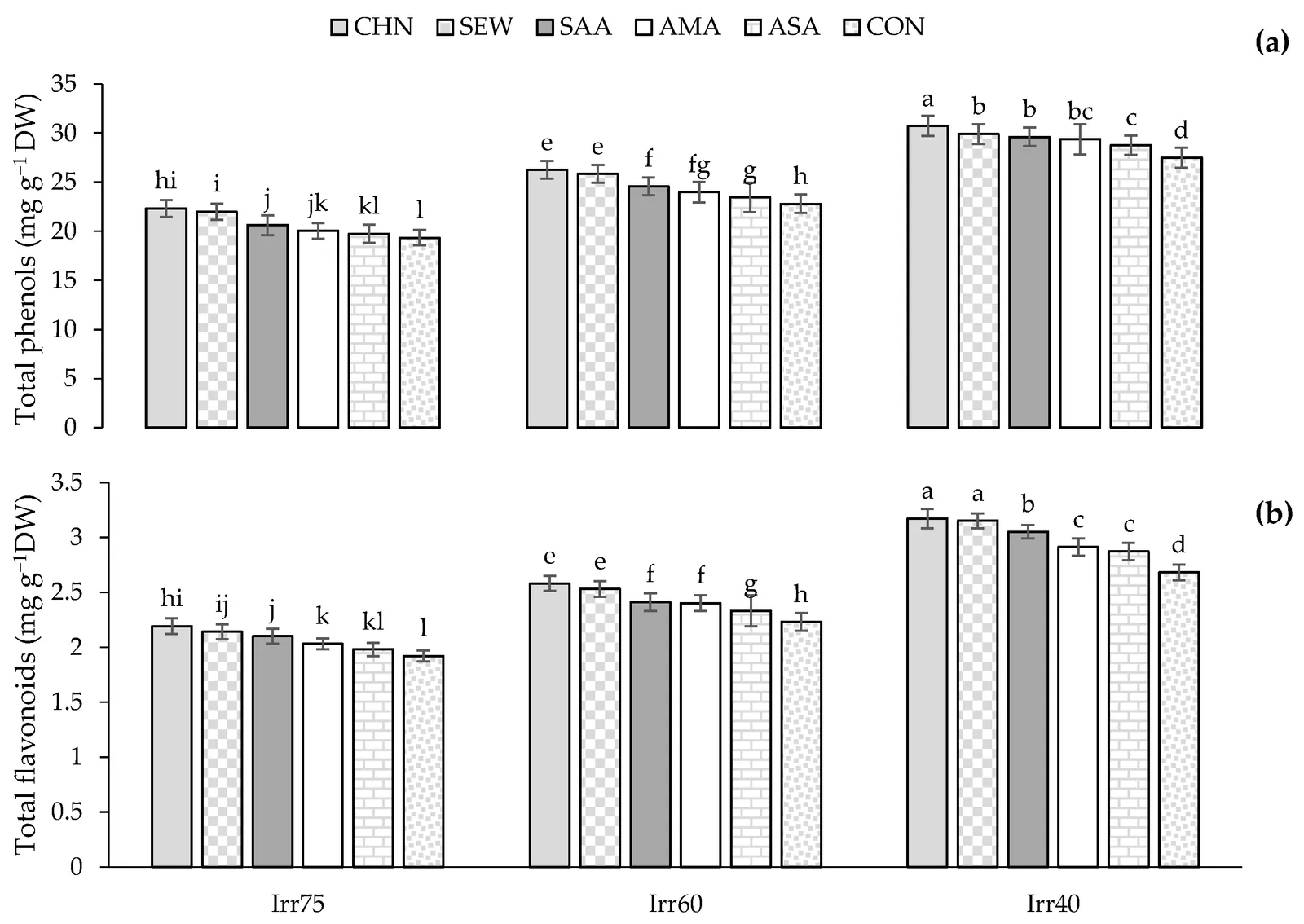
Means comparison and the interactive influence of stress modulators and irrigation regime use on TPC (**a**) and TFC (**b**). Similar letters indicate non-significant difference by LSD test at *p* ≤ 0.05. CHN, chitosan; AMA, amino acids; SEW, seaweed; ASA, ascorbic acid; SAA, salicylic acid; CON, control; Irr40, 40, Irr60, 60, and Irr75, 75% field capacity.

The average TPC and TFC of oregano plants were 25.61 and 2.54 mg g^−1^ DW in 2018 and 24.02, and 2.43 mg g^−1^ DW in 2019, respectively (Table 2). The TPC and TFC in Irr75 and Irr60 plants were higher than in Irr40 (Table 2). Finally, among the stress modulators, CHN favored the highest TPC and TFC (Table 2). TPC and TFC achieved the maximum values (30.72 and 3.17 mg g^−1^ DW) in plants treated with CHN under Irr40 and the lowest ones (1.92 and 19.33 mg g^−1^ DW) in control plants under Irr75 (Figure 7a,b).

#### 2.2.4. Nutrients of N, K, and P

**Figure 8:**
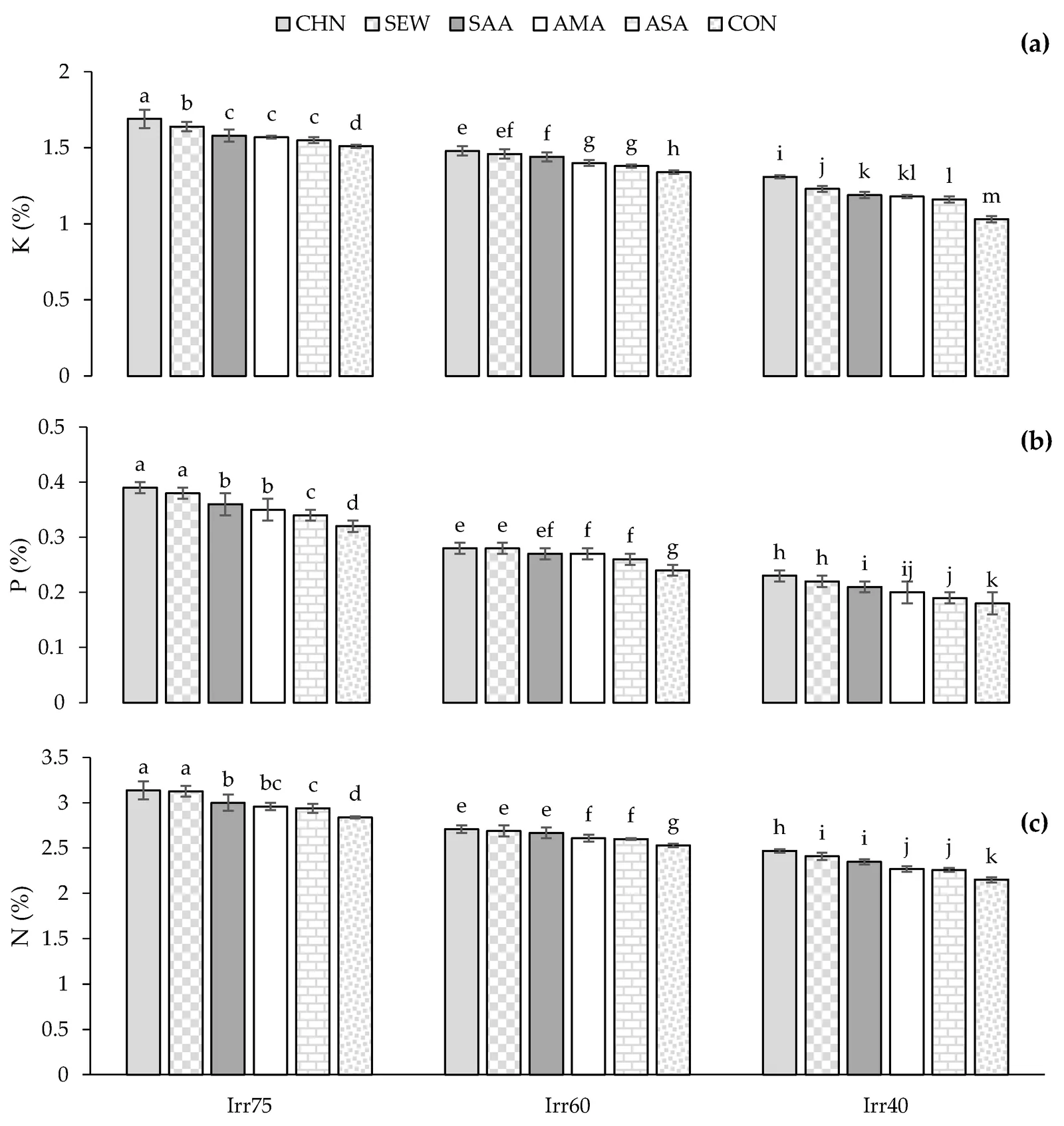
Means comparison and the interactive influence of stress modulators and irrigation regime use on the K (**a**), P (**b**), and N (**c**). Similar letters indicate non-significant difference by LSD test at *p* ≤ 0.05. CHN, chitosan; AMA, amino acids; SEW, seaweed; ASA, ascorbic acid; SAA, salicylic acid; CON, control; Irr40, 40, Irr60, 60, and Irr75, 75% field capacity.

The percentage of N, P, and K were significantly higher in Irr75 than in Irr60 and Irr40 (Table 2). The application of stress modulators increased the percentage of N, P, and K significantly versus control plants (Table 2). The major concentrations of N, P, and K (3.14, 0.39, and 1.69%, respectively) were obtained in plants treated with CHN under Irr75, whereas the lowest (1.03, 0.18, and 2.15%) in control plants under Irr40 (Figure 8a–c).

## 3. Discussion

Moisture shortage stress has a detrimental effect on plants, as it reduces cell viability and hinders cell development, division, and elongation, leading to a decrease in vegetative and morphological growth. Under moisture shortage stress, the stomatal conductance decreases due to increased resistance to the release of carbon dioxide, caused by the inhibition of Rubisco activity, which can result in an increase in the water potential of the crop [2]. The observed reduction in dry weight yield with increasing water stress may be ascribed to the preferential allocation of resources to the roots due to soil moisture limitations or a lower chlorophyll content, thereby reducing photosynthetic efficiency [19]. As a result of the moisture shortage stress, plants attempt to minimize transpiration by closing their stomata, leading to decreased gas exchanges [20], lower CO_2_ intake, and increased oxygen levels, ultimately reducing the carboxylation activity of Rubisco, and, consequently, dry matter production [19]. Conversely, the foliar spraying of CHN, AMA, SEW, ASA, and SAA enhanced the total dry weight (TDW) of oregano plants under both favorable irrigation conditions and water deficit. This positive effect can be attributed to the expansion of leaf surface area which improved leaf durability and enhanced the utilization of light resources [21]. AMA, SEW, ASA, and SAA were indeed shown to enhance crop development, growth, and production under moisture shortage stress by improving nutrient availability and absorption [7,22]. These biostimulants effectively contribute to maximizing the chelation, transport, and absorption of available nutrients from the soil and are already rich in plant growth regulators that promote the production of new structural biomass. For instance, Shafie et al. [23] stated that foliar application of AMA, and SEW improved the photosynthetic activity, plant development, and growth. Similarly, the stimulating effect of stress modulators like AA and SEW on plant growth is attributed to the increased content of endogenous plant growth-promoting hormones [7].

Foliar application of AMA, SEW, ASA, SAA, and, most of all, CHN, not only favors the rate at which plant utilizes CO_2_, but also affects the evaporation/transpiration ratio, the synthesis of growth stimulants, the leaf water potential, root development, and the water uptake of oregano [14,22,24].

Biostimulant application is also reported to boost the cell membrane permeability, facilitating potassium entry and increasing intracellular pressure, cell division, and production [19,20]. This study demonstrated that the percentage of oregano EOY raised with increasing water shortage regimes and such an effect was emphasized in combination with the foliar application of AMA, SEW, ASA, SAA, and CHN [7].

It is known that drought stress triggers the production of secondary metabolites and essential oils, which serve as protective molecules against environmental stresses [7,25,26]. More specifically, essential oils are terpenoids that require acetyl-CoA, NADPH, and ATP for their synthesis [27]. Therefore, the production of these compounds is closely linked to the plant’s nutrient availability [19]. In severe stress conditions, the plant allocates most of the produced photosynthetic resources to the synthesis of osmoregulating compounds like glycine betaine, proline, and sugar such as sucrose, fructose, and fructans, rather than allocating resources to growth [28]. This process contributes to creating the necessary conditions for the survival of plants under more severe stress conditions [4]. The increase in essential oil under moisture shortage stress can be explained as follows: as the leaf surface diminishes due to water stress, the density of essential oil-secreting glands increases, leading to the accumulation of essential oil in leaf tissue. Consequently, this accumulation has a direct impact on the overall EOY production [29]. The biostimulant composition in terms of macro and microelements, vitamins, amino acids, and growth hormones, including cytokinin, plays a crucial role in promoting plant growth and the percentage and yield of essential oil. Therefore, the foliar spraying of biostimulants contributed to maximizing such an effect [27,30], likely activating different biosynthetic pathways and genes responsible for secondary metabolite production and enhancing the glands producing essential oils in leaves and flowers of the oregano plants under water stress. The alteration in the EOY due to the action of biostimulants is also probably ascribed to the increased CO_2_ assimilation [25]. Since leaves are the most important metabolic organ, the foliar application may act as the most effective method to influence plant metabolism. In fact, there are several studies reporting the effects of enzymes and peptides associated with the formation of secondary metabolites in medicinal and aromatic plants in response to biostimulant treatments [25,26].

Reduced growth and root activity, coupled with increased evaporation and transpiration from the crop area, have been reported as important factors contributing to the decline in RWC [8]. The decrease in leaf RWC due to severe irrigation regimes is primarily due to the reduced water absorption by the roots and increased water transpiration through the leaves, ultimately leading to the closure of leaf stomata [31]. The decline in the plant tissue turgor and RWC can represent an initial sign of stress having a direct influence on cell division and size [23]. However, the foliar application of AMA, SEW, ASA, SAA, and, most of all, CHN, significantly improved the RWC in all irrigation regimes, with a positive impact on root development, evaporation/transpiration ratio, leaf water potential, and the water uptake of oregano plants [14,22,24].

The reduction in Chl content under conditions of moisture shortage stress can be ascribed to the water deficiency and the production of reactive oxygen species responsible for the decomposition and peroxidation of chlorophylls. ROS are known to destroy lipids and proteins, decrease the stability of the chloroplast membranes, leading to their breakdown, and reduce the content of photosynthetic pigments [14,20,24]. However, both under optimal irrigation regimes and water deficit, the foliar application of CHN, AMA, SEW, ASA, and SAA enhanced the concentration of Chl a and Chl b in oregano plants. These biostimulants, play a crucial role in cell division and growth, plant nutritional cycle, electron transport system, stomatal conductance, and transpiration rates [22,23]. They are rich in plant growth regulators such as gibberellins, auxins, and cytokinins, which stimulate the activation of enzymes involved in photosynthesis and pigment production [32,33,34,35]. Biostimulants are also reported to influence the chloroplast size [22,23], and the number of chloroplasts within the cells, which in turn promote the synthesis of more chlorophylls [24]. Moreover, given the strong correlation between chlorophyll content and nitrogen concentration, it is possible that adequate nitrogen availability resulting from the application of the stress modulators ensured plants a sufficient nitrogen supply for Chl production.

Proline is the most abundant and recognized soluble organic osmolyte that accumulates in response to moisture shortage stress providing protecting functions on cytosol enzymes and cell structure [10,36]. The accumulation of water deficit can depend on different factors: some studies related this response to the regulatory effect of ABA on the light-dependent processes involved in proline synthesis [37], while others have attributed this response to higher-energy photosynthetic compounds that promote the mechanism of proline synthesis [8]. Proline accumulation is considered the main osmotic adjustment mechanism in enhancing the stress tolerance of crops [37]. Plants sprayed with stress modulators such as SAA and SEW usually exhibit more efficient nutrient and water management compared to untreated ones under moisture shortage stress. Therefore, the proline accumulation in oregano plants tends to increase to a lesser extent when stress modulators are applied. Specifically, stress modifiers, through their hormonal effects, increase the number of root branches, influencing plant cell metabolism and chelating capabilities, and enhancing nutrient and water absorption, resulting in the reduction of proline concentration [7,14,15,22,30].

The delay in irrigation enhanced the concentration of TSS in oregano leaves, representing a potential strategy for plant growth in low water conditions. The principal source of TSS is the hydrolysis of carbon reserves [34]. Since both photosynthesis and growth are affected by drought stress, the accumulation of TSS is influenced by their balance [33]. Considering that growth is affected before photosynthesis may be impaired by water shortage, the accumulation of photosynthesis products can be expected [11]. Therefore, sucrose acts as a signaling molecule in low concentrations and as a scavenger of reactive oxygen species in high concentrations [38]. The soluble sugars, playing the role of osmotic regulators, are involved in the regulation of osmosis and the reduction of the water potential. Therefore, they help plants retain more water inside cells and maintain turgor under drought stress, increasing plant resistance to cope with water deprivation conditions [38,39]. Higher levels of soluble sugars were observed in plants treated with stress modulators, as the foliar application of CHN, AMA, SEW, ASA, and SAA, probably enhanced vegetative growth and led to a greater concentration of carbohydrates [26,33]. The increase in TSS may also depend on the up-regulation of different genes involved in sugar transport and metabolism [30]. Furthermore, also in this case, the higher concentration of sugars likely contributed to enhancing drought resistance by improving osmotic adjustment and plant response to dehydration stress by maintaining carbon balance [26]. Substances like SAA and ASA, which can remove hydrogen peroxide, are effective in preventing pore closure and mitigating oxidation damage caused by ROS accumulation, particularly on Calvin cycle enzymes. Therefore, SAA and ASA can improve photosynthesis and the synthesis of carbon products [40]. The increase in TSS in stressful conditions, which affects the osmotic potential, helps to maintain the health and function of cell membranes that may have been damaged by water stress.

The activity of CAT, APX, and SOD enzymes was raised in oregano plants with increasing water shortage, consistent with Rahimi et al. [7], who reported that in garden thyme plants, the water stress triggered the activation of antioxidant enzymes. There is indeed a strong correlation between moisture shortage stress and plant capability to activate antioxidant defenses [39]. The improvement of antioxidant enzyme activities is a plant defense mechanism against various biotic and abiotic stresses. Under moisture shortage stress, ROS accumulating in plant cells can damage lipids, nucleic acids, carbohydrates, and membranes and can trigger metabolic alterations [9,39]. To detoxify and eliminate ROS, plant cells employ an enzymatic defense strategy, which upsurges the tolerance to moisture shortage stress [41]. More specifically, crops synthesize and recruit the antioxidant enzymes CAT and SOD to remove the excess ROS and activate their immune system [42,43]. Under water deficit, SOD is the initial defense against ROS deterioration by converting the O_2_^−^ into H_2_O_2_ and O_2_. Even though small concentrations of H_2_O_2_ are beneficial, acting as an intracellular signal, at high concentrations, H_2_O_2_ can damage cells [44]. Thus, at this stage, the CAT enzyme catalyzes the conversion of H_2_O_2_ into H_2_O and other non-toxic products [42]. APX and CAT are important antioxidant enzymes that transform “H_2_O_2_” into H_2_O and detoxify cells from ROS during stressful situations [39]. By reducing soil moisture content, CAT, SOD, and APX activities increased in oregano plants treated with the foliar application of the stress modifiers, according to previous findings, evidencing the increasing plant tolerance to water deficit after receiving biostimulant treatments [7,34,35]. The mechanism by which CHN neutralizes free radicals may be related to its structure, which consists of a large number of accessible amine and hydroxyl groups that can react with free radicals [33,45]. These results suggest that stress modifiers may help mitigate oxidative stress by maintaining steady-state intercellular ROS levels, and, consequently, protecting cell membranes from damage caused by a severe water deficit [23,33,43,45]. Stress modifiers can also influence the resistance to water deficit and adverse environmental factors by affecting the expression of specific plant genes involved in the defense mechanisms against stressful conditions [7].

In addition to stimulating the antioxidant activities of CAT, SOD, APX, and POX enzymes, the modifiers CHN, AMA, SEW, ASA, and SAA induced also the rise of phenolic compounds [33,34,35]. Phenols and flavonoids are secondary natural metabolites, serving as scavengers against free radicals and oxidative species protecting cytoplasmic and chloroplastic structures and preventing the oxidation of lipids from the action of lipoxygenase [21,46]. The results indicate that by increasing the irrigation intervals, the content of phenols and flavonoids in oregano plants started rising. Moreover, even under severe deficit irrigation conditions, with the foliar application of CHN, AMA, SEW, ASA, and SAA, phenols and flavonoids achieved their maximum quantities [23,47,48,49]. The application of stress modulators to reduce the effect of free radicals increasing the synthesis of phenolic compounds has been proven to be highly beneficial under water deficit [50]. Such an effect may be related to the role of CHN, AMA, SEW, ASA, and SAA in enhancing antioxidants active against ROS [7,47,48].

Foliar applications of CHN, SEW, ASA, and SAA are reported to increase the photosynthesis ratio and the activity of enzymes involved in starch biosynthesis and the production of secondary metabolites [23,51]. Since hydrocarbons are required as a skeleton to synthesize phenols, an augment in their concentration results in the accumulation of phenolic compounds. Such increase may be ascribed to the allocation of more carbon resources to the shikimate pathway, particularly contributing to the production of flavonoids and phenols [50] useful to enhance plant tolerance to biotic and abiotic stresses [23,51].

When soil water availability is limited, the uptake of water, the solubility of nutrients in the soil, and their absorption by the roots decrease with negative consequences on photosynthesis and transpiration rates [9,31]. Consequently, plants reduce their growth to compensate for the water deficiency and lack of nutrients as observed in thyme plants under water stress [9,46]. Under moisture shortage stress conditions, plants experience water scarcity and osmotic stress due to a decrease in soil matrix potential, resulting in ionic imbalances and nutrient deficiency [52]. In these conditions, as previously demonstrated in other species like basil, the foliar application of CHN, AMA, SEW, ASA, and SAA positively affects stomatal regulation and chlorophyll concentration, stimulates the division and lengthening of root cells promoting the expansion of the radical apparatus, and, consequently, improving the water and nutrient uptake and plant vegetative growth. Finally, biostimulants, and in particular chitosan, play an important signaling role in the activation of different plant defense responses like the biosynthesis of special secondary metabolites [23,51] and the activation of antioxidant enzymes, protecting plants even under water shortage [7,30,33].

## 4. Materials and Methods

### 4.1. Experimental Design

The research was conducted at the study farmstead of the Medicinal Plants and Drugs Research Institute of the Urmia University located in West Azerbaijan, Iran (45°10′ E, 37°44′ N, and 1338 m. above sea level). This research lasted two years (2018 and 2019) and was designed as a factorial experiment based on a randomized complete block design with five stress modulators treatment levels (CHN: chitosan, AMA: amino acids, SEW: seaweed, ASA: ascorbic acid, SAA: salicylic acid, and CON: control) and irrigation regime at three levels (40 (Irr40), 60 (Irr60), and 75 (Irr75) % field capacity) in three replications.

At the beginning of flowering, three levels of irrigation regime (the plots were irrigated to 100% field capacity after depletion 40% (Irr40), 60% (Irr60), and 75% (Irr75) of soil available water, respectively) were exerted through controlling water application. Soil moisture content in each plot was monitored daily using a Time Domain Reflectometry device (TRIM-FM TDR 10776, Weilheim, Germany).

At both regions, TDR tubes were placed in each plot to determine soil moisture content in the top layer of soil (0–30 cm). Maximum Allowable Depletion (MAD) of available soil water (ASW) in the effective root zone was determined using Equation (1) [53]:

MAD = FC − θ/FC − PWP(1)

where FC is the soil volumetric moisture at field capacity, θ is the soil volumetric moisture and PWP is the soil volumetric moisture at permanent wilting point. The required volume of water (Vd, mm) was estimated according to Equations (2) and (3) [54]:

ASW = FC − PWP(2)

**Figure 9:**
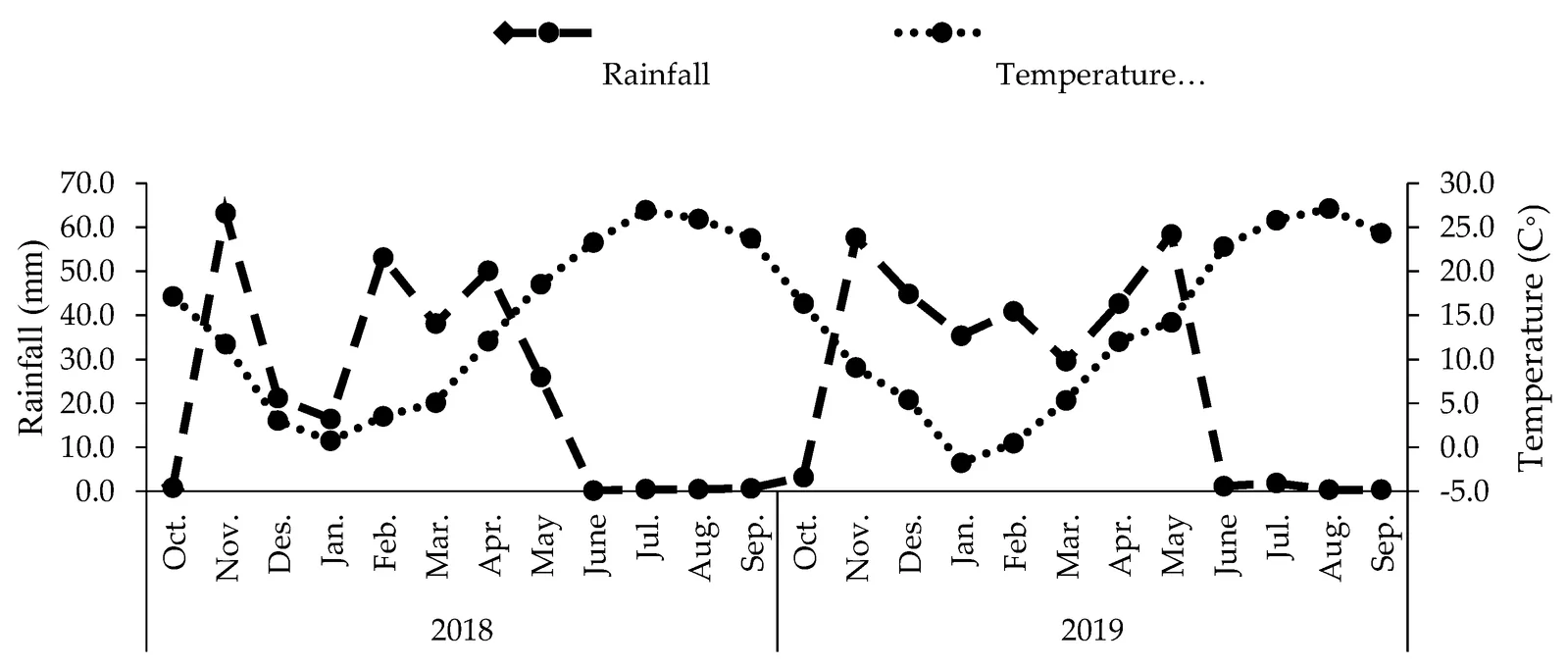
Total rainfall and average monthly air temperature for the 2018 and 2019 growing seasons.

Vd = MAD × ASW × Rz × 10(3)

where ASW is equal to 15.67 cm m^−1^, Rz is the effective rooting depth (0.4 m), and 10 is the conversion constant from cm to mm. The furrow system was used for irrigation. Each plot was watered individually, and a hose (4 cm diameter) with a gauge was used to adequately convey the required volume of irrigation water. Monthly rainfall and air temperature as average are shown in Figure 9.

### 4.2. Plant Material and Stress Modulators Application

The seeds of *O. vulgare* L. were provided by the Pakan Bazr Seed Company. The seeds of *O. vulgare* L. were primarily planted in seedling plates with a blend of soil: perlite (2:1, *v*:*v*) in March 2017. Each experimental unit consisted of five rows of planting, each four meters in length, with oregano seedlings that were 8 cm tall, planted on 5 May in a plant spacing of 50 × 25 cm in the farms.

The foliar spray treatments contained a foliar use of amino acids (2 mL L^−1^: Agri Tecno Co., Valencia, Spain), seaweed (2 mL L^−1^; Acadian AgriTech Co., Dartmouth, NS, Canada), ascorbic acid (2 mL L^−1^; Sigma-Aldrich Co., Barcelona, Spain), salicylic acid (2 mL L^−1^; Sigma-Aldrich Co., St. Louis, MO, USA), and Chitosan (2 mL L^−1^; Sigma-Aldrich Co., Steinheim, Germany), in addition to the control plants which were sprayed with pure water. The soil physicochemical characteristics measured were pH (7.9), electrical conductivity (0.48 dS/m), soil texture (clay silty, silt 36%, clay 33%, sand 31%), total nitrogen (0.095%), phosphorus (7 mg/kg), and potassium (298 mg/kg). Foliar spraying was performed in the vegetative phase of oregano, simultaneously with moisture shortage stress, in three steps at a ten-day interval. Weeds were manually managed when required.

### 4.3. Measurements

#### 4.3.1. Total Dry Weight (TDW) Determination

The measured growth parameter was the TDW per plant at the maximum flowering phase from 8 cm of the soil surface. The plant samples of oregano were oven dried at 70 °C for 48 h and, after, reweighed on a scale.

#### 4.3.2. Essential Oil Yield (EOY)

The EOY of *O. vulgare* L. plants was extracted by the Clevenger method [55], using distilled water and an EO extractor. For this purpose, 50 g of an *O. vulgare* L. dry matter mix composed of leaf and flower material was weighed from each plot, and ground to pass a 1 mm screen. The ground samples were then placed in a jar with 500 mL of water and boiled inside Clevenger for 3 h to extract its essential oil. The yield of EO was calculated using Equation (4), where M is the mass of the extracted oil (g) and Bm is the initial plant biomass (g) [56]:

(4) EOY=(M/Bm)×100

#### 4.3.3. Relative Water Content (RWC)

The RWC was performed agreeing to the following Equation (5) [57]:

% RWC = [(FW − DW)/(TW − DW)] × 100(5)

After fresh weight (FW) determination, the leaves were soaked in distilled water for 16 to 18 h. The turgid samples were quickly blotted dry to remove any excess surface water and then, the turgid weight (TW) was determined. The dry weight (DW) of the samples was obtained after being oven dried at 70 °C for 24 h.

#### 4.3.4. Chl a and Chl b Content

The content of Chl a and Chl b was assessed on a weight basis by extracting the fresh leaves using 80% acetone. The concentration of Chl a and Chl b was determined spectrophotometrically according to Equations (6) and (7) [58].

Chl a = 11.24 × A662 − 2.04 × A645(6)

Chl b = 20.13 × A645 − 4.19 × A662(7)

#### 4.3.5. Proline Content

Proline content was performed by ninhydrin reagent protocol following the procedure reported in Paquin and Lechasseur [59], using 0.5 g of fresh leaf. The absorbance was evaluated at 515 nm by Spectronic 20 colorimeter (SP 6-200 Unican Montréal-Est, Quebec, QC, Canada). L-proline was used as standard to make a reference curve (R^2^ = 0.99, ranging from 0 to 150 mg L^−1^). Proline content was established from the reference curve and expressed as mg g^−1^ of FW.

#### 4.3.6. Total Soluble Sugars (TSS) Determination

Leaf TSS was performed, as founded by the phenol-sulfuric acid methodology [60]. In this manner, 0.5 g of fresh leaves was homogenized with ethanol. The extract was filtered and treated with 5% phenol and 98% sulfuric acid. This mixture was left for 1 h and its absorption was measured by spectrophotometer at 625 nm. For TSS determination a standard curve (R^2^ = 0.99, ranging from 0 to 80 mg L^−1^) was produced by plotting solutions with varying D-glucose concentrations. The results were assessed through the standard curve and expressed as mg g^−1^ of FW.

#### 4.3.7. Enzyme Extractions and Assays

To quantify the antioxidant enzyme activity, fresh material (100 mg) was ground in 2 mL of 0.1 M KH_2_PO_4_, containing 5% polyvinylpyrrolidone (PVP), and buffered at a pH of 6. Thereafter, the extracts were centrifuged at 3 °C for 30 min at 15.000 rpm, and the enzyme activity was estimated using the supernatant [61].

Catalase (CAT) activity was quantified at 240 nm based on the variation of concentration of hydrogen peroxide (H_2_O_2_). In this case, the reaction mixture contained 1.9 mL of 50 mM K_3_PO_4_, which was buffered at a pH of 7, 10 mM H_2_O_2_, and 0.2 mL of enzyme extract. Enzymatic activity was then read in 60 s per mg of protein based on absorption variations [62].

Superoxide dismutase (SOD) activity was assessed at 560 nm to minimize the loss of nitroblue tetrazolium (NBT) photochemical as noted by Beyer and Fridovich [63]. In this study, one unit of SOD was taken as the quantity of enzyme that inhibits a 50% decrease in NBT.

The ascorbate peroxidase (APX) activity was measured with the Nakano and Asada [64] using a reaction mixture containing 1 mL of 0.5 mM ascorbic acid, and 1 mL 100 mM K_3_PO_4_ buffered at a pH of 7, 100 μL enzyme extract, and 0.1 mL H_2_O_2_ 0.1 mM. The absorption was then read at 290 nm.

#### 4.3.8. Total Polyphenols and Flavonoids (TPC and TFC)

The TPC was evaluated using the Folin–Ciocalteau technique [65,66]. An amount of 1600 μL of distilled water and 10 μL of methanolic extracts were mixed and treated with 200 μL of Folin–Ciocalteau reagent (10% *v*/*v*), which was prepared in distilled water for 5 min at 25 °C. Thereafter, 200 μL of NaCO_3_ (7.5%) was added to the mixture and kept at 25 °C in the dark for 30 min. For the quantitative estimation of TPC, the sample’s absorbance was measured using a UV/visible spectrophotometer (DB-20/DB-20S, Dynamica, UK) at 760 nm. The calibration curve (R^2^ = 0.9556) with gallic acid, ranging from 0 to 500 mg L^−1^, was used to determine the TPC. The results are presented in milligrams of equivalent gallic acid (3,4,5 trihydroxybenzoic acid) per gram of sample dry weight (mg GAE g^−1^ DW).

The aluminum chloride-based colorimetric method was used to evaluate the TFC in flower extracts. In brief, 150 μL of sodium nitrate (5% *w*/*v*) was mixed with 30 μL of the extract. After waiting for 5 min, 3 mL of aluminum chloride hexahydrate (10% *w*/*v*) was added to the reaction mixture, and incubated for 5 min. Thereafter, 1 mL of NaOH (1.0 M) was added followed by a proper dilution with distilled water.

After incubation in the dark for 30 min at 25 °C, the solution’s absorbance was measured in a spectrophotometer at 510 nm. The external standard for TFC quantification was Quercetin (QE). The TFC was determined using a quercetin standard curve (R^2^ = 0.9626) ranging from 0 to 0.03 mg L^−1^. The results are given as milligrams of equivalents for each gram of dry weight of the samples (mg QE g^−1^ DW) [65,66].

#### 4.3.9. Nutrients of N, P and K

To measure nutrient uptake, oregano leaf samples were milled, digested, and examined via combustion (4 h at 500 °C). The ashes (5 mg) were digested in 1 mL of 2 N HCl, and with the help of Whatman filter paper: grade 42, the extracts were purified. With the help of the vanadomolybdate technique, the phosphorus (P) uptake was performed calorimetrically. The process was established on the subordination of the yellow color of the unreduced vana-do-molybdo-phosphoric heteropoly acid balanced in an HNO_3_ medium. The color intensity was decided at 470 nm utilizing a Spectronic 20 colorimeter (Montréal-Est, Quebec, QC, Canada) [67,68]. The quantification of P content was carried out with a calibration curve (R^2^ = 0.9991) in the range 0–0.4 mg L^−1^. The results are given as percentages (%). The uptake of potassium (K) was performed by a flame photometer (Jenway, UK) [67,68] and its amount was estimated by a standard curve (R^2^ = 0.985) in the range 0–0.45 mg L^−1^. The results are given as percentages (%). The nitrogen (N) was performed using the Kjeldahl technique (Wertheim, Germany) [69].

### 4.4. Data Analysis

A two-year analysis of variance (ANOVA) was performed using the general linear model (GLM) (SAS 9.1.3) combined the results from the years 2018 and 2019. The effects of irrigation regime, the application of stress modifier biostimulants, and the interactions between these two variables were evaluated by ANOVA and distinctions between means were compared using the LSD test at *p* < 0.05.

## 5. Conclusions

Moisture shortage stress-induced negative effects on oregano plants, reducing total dry matter, Chl a and b content, and nutrient uptake (N, P, and K). However, it increased the essential oil yield, proline, total soluble sugars, and total phenols, and the antioxidant activity of several enzymes. The application of chitosan (CHN) was effective in mitigating the negative impacts of moisture shortage stress by increasing total dry matter, chlorophyll a and b content, osmotic adjustment, and antioxidant activity and stimulating the production of essential oil yield. Similar positive responses were also observed when applying the seaweed (SEW) stress modulator, suggesting that CHN and SEW significantly helped the oregano plant to withstand water stress through higher proline levels and osmotic adjustments. Overall, among all the tested biostimulants, CHN is recommended as a source of valuable substances beneficial for the growth and development, physiological traits, and essential oil production of oregano plants, reducing the harmful consequences of moisture shortage stress and providing a sustainable solution for oregano production even under water stress conditions.

## Figures and Tables

**Table 1:** Analysis of two-year variance and comparison of means of TDW, EOY, RWC, Chl a and b traits of oregano plants in response to irrigation regime (Irr), stress modifier biostimulants (Str), and their interaction (Irr × Str). Different letters in each column indicate significant differences according to LSD test (*p* < 0.05).

Source of Variation	df	TDW (g m^−2^)	EOY (%)	RWC (%)	Chl a (mg g^−1^ FW)	Chl b (mg g^−1^ FW)
Year (Y)	1	201.93 *	0.0003 ^ns^	65.27 **	0.0003 ^ns^	0.0001 ^ns^
Rep (Y)	4	0.50	0.001	0.24	0.10	0.007
(Irr)	2	10,483.58 **	0.43 **	3128.56 **	8.85 **	4.58 **
Y × Irr	2	6.94 ^ns^	0.0009 ^ns^	1.93 ^ns^	0.0000009 ^ns^	0.00003 ^ns^
(Str)	5	2119.89 **	0.31 **	619.68 **	0.82 **	0.19 **
Y × Str	5	2.28 ^ns^	0.0002 ^ns^	0.84 ^ns^	0.00002 ^ns^	0.00005 ^ns^
Irr × Str	10	644.55 **	0.07 **	184.34 **	0.19 **	0.08 **
Y × Irr × Str	10	2.81 ^ns^	0.0004 ^ns^	0.45 ^ns^	0.00004 ^ns^	0.00003 ^ns^
Error	68	21.17	0.006	6.83	0.01	0.002
CV (%)		4.15	1.64	4.16	4.82	3.54
**Y**						
2018		109.35 ± 19.81 ^b^	1.54 ± 0.17 ^a^	61.92 ± 10.73 ^b^	2.22 ± 0.49 ^a^	1.37 ± 0.30 ^a^
2019		112.08 ± 18.79 ^a^	1.53 ± 0.18 ^a^	63.48 ± 10.26 ^a^	2.23 ± 0.51 ^a^	1.38 ± 0.32 ^a^
**Irr**						
Irr75		132.79 ± 7.05 ^a^	1.33 ± 0.06 ^c^	72.66 ± 7.05 ^a^	2.71 ± 0.15 ^a^	1.76 ± 0.11 ^a^
Irr60		109.14 ± 6.68 ^b^	1.54 ± 0.05 ^b^	61.25 ± 8.60 ^b^	2.25 ± 0.29 ^b^	1.30 ± 0.16 ^b^
Irr40		90.21 ± 6.62 ^c^	1.73 ± 0.06 ^a^	54.19 ± 5.75 ^c^	1.72 ± 0.32 ^c^	1.06 ± 0.14 ^c^
**Str**						
CHN		115.94 ± 18.90 ^a^	1.57 ± 0.18 ^a^	71.17 ± 8.74 ^a^	2.49 ± 0.60 ^a^	1.53 ± 0.36 ^a^
SEW		113.28 ± 15.63 ^ab^	1.55 ± 0.21 ^ab^	68.02 ± 5.69 ^b^	2.39 ± 0.49 ^b^	1.41 ± 0.33 ^b^
SAA		112.99 ± 20.06 ^ab^	1.55 ± 0.12 ^b^	63.18 ± 8.13 ^c^	2.28 ± 0.22 ^c^	1.41 ± 0.24 ^b^
AMA		112.05 ± 17.19 ^b^	1.54 ± 0.18 ^b^	59.58 ± 8.10 ^d^	2.20 ± 0.33 ^d^	1.35 ± 0.23 ^c^
ASA		108.61 ± 21.71 ^c^	1.53 ± 0.15 ^c^	57.41 ± 6.02 ^e^	2.11 ± 0.53 ^e^	1.31 ± 0.37 ^d^
CON		101.42 ± 16.68 ^d^	1.44 ± 0.19 ^d^	56.86 ± 4.77 ^e^	1.89 ± 0.44 ^f^	1.23 ± 0.32 ^e^

**, *, and ns, significant at 1% and 5% levels of probability, non-significant, respectively. TDW, total dry weight; EOY, essential oil yield; RWC, relative water content; Chl a and b, chlorophyll a and b content.

**Table 2:** Analysis of two-year variance and comparison of means of Pro and TSS content, TPC and TFC, CAT, SOD, and APX activity, N, P, and K amount in oregano plants in response to irrigation regime (Irr), stress modifier biostimulants (Str), and their interaction (Irr × Str). Different letters in each column indicate significant differences according to LSD test (*p* < 0.05).

Source of Variation	df	Pro (mg g^−1^ FW)	TSS (mg g^−1^ FW)	CAT (μmol min^−1^ g^−1^ FW)	SOD (μmol min^−1^ g^−1^ FW)	APX (μmol min^−1^ g^−1^ FW)	TPC (mg g^−1^ DW)	TFC (mg g^−1^ DW)	N (%)	P (%)	K (%)
Year (Y)	1	1.60 **	3.93 ^ns^	0.74 **	61.59 **	0.03 **	67.97 **	0.31 **	0.002 ^ns^	0.00002 ^ns^	0.00001 ^ns^
Rep (Y)	4	0.02	0.48	0.01	0.24	0.002	1.14	0.004	0.005	0.0003	0.001
(Irr)	2	14.31 **	1471.36 **	6.64 **	2459.59 **	0.57 **	572.20 **	6.30 **	3.38 **	0.12 **	1.28 **
Y × Irr	2	0.005 ^ns^	0.34 ^ns^	0.30 **	2.47 ^ns^	0.001 ^ns^	0.22 ^ns^	0.005 ^ns^	0.0006 ^ns^	0.000003 ^ns^	0.0004 ^ns^
(Str)	5	1.16 **	14.81 **	2.25 **	452.3 **	0.07 **	45.95 **	0.63 **	0.25 **	0.02 **	0.10 **
Y × Str	5	0.00003 ^ns^	0.01 ^ns^	0.05 ^ns^	0.82 ^ns^	0.0002 ^ns^	0.0001 ^ns^	0.0002 ^ns^	0.001 ^ns^	0.000005 ^ns^	0.0001 ^ns^
Irr × Str	10	0.28 **	13.57 **	0.92 **	175.94 **	0.05 **	10.99 **	0.11 **	0.13 **	0.008 **	0.03 **
Y × Irr × Str	10	0.00001 ^ns^	0.009 ^ns^	0.06 ^ns^	0.32 ^ns^	0.0001 ^ns^	0.00004 ^ns^	0.0001 ^ns^	0.0008 ^ns^	0.000008 ^ns^	0.0002 ^ns^
Error	68	0.006	1.27	0.04	6.83	0.0008	0.29	0.002	0.002	0.0001	0.0006
CV (%)		2.56	6.61	9.52	5.51	3.18	2.17	2.03	1.81	3.83	1.82
**Y**											
2018		3.34 ± 2.13 ^a^	16.87 ± 5.49 ^a^	2.37 ± 2.13 ^a^	48.17 ± 9.66 ^a^	0.95 ± 0.14 ^a^	25.61 ± 3.83 ^a^	2.54 ± 0.41 ^a^	2.65 ± 0.30 ^a^	0.26 ± 0.05 ^a^	1.39 ± 0.18 ^a^
2019		3.10 ± 0.59 ^b^	17.25 ± 5.57 ^a^	2.20 ± 2.15 ^b^	46.66 ± 9.18 ^b^	0.91 ± 0.13 ^b^	24.02 ± 3.72 ^b^	2.43 ± 0.40 ^b^	2.68 ± 0.28 ^a^	0.28 ± 0.06 ^a^	1.42 ± 0.19 ^a^
**Irr**											
Irr75		2.58 ± 0.38 ^c^	10.54 ± 1.89 ^c^	1.89 ± 0.46 ^c^	39.81 ± 6.85 ^c^	0.81 ± 0.12 ^c^	20.75 ± 2.38 ^c^	2.09 ± 0.27 ^c^	2.97 ± 0.17 ^a^	0.34 ± 0.04 ^a^	1.58 ± 0.09 ^a^
Irr60		3.23 ± 0.32 ^b^	17.32 ± 1.57 ^b^	2.21 ± 0.47 ^b^	46.21 ± 7.49 ^b^	0.91 ± 0.10 ^b^	24.98 ± 2.02 ^b^	2.43 ± 0.21 ^b^	2.62 ± 0.19 ^b^	0.27 ± 0.05 ^b^	1.41 ± 0.09 ^b^
Irr40		3.84 ± 0.25 ^a^	23.32 ± 1.62 ^a^	2.74 ± 0.55 ^a^	56.21 ± 5.23 ^a^	1.06 ± 0.07 ^a^	28.72 ± 1.62 ^a^	2.92 ± 0.15 ^a^	2.36 ± 0.13 ^c^	0.22 ± 0.04 ^c^	1.20 ± 0.10 ^c^
**Str**											
CHN		2.82 ± 0.56 ^e^	18.50 ± 5.29 ^a^	2.81 ± 0.51 ^a^	53.88 ± 9.13 ^a^	1.02 ± 0.15 ^a^	26.44 ± 3.48 ^a^	2.68 ± 0.37 ^a^	2.83 ± 0.26 ^a^	0.32 ± 0.05 ^a^	1.51 ± 0.15 ^a^
SEW		3.05 ± 0.57 ^d^	17.51 ± 4.96 ^b^	2.63 ± 0.19 ^b^	52.30 ± 4.88 ^a^	1.00 ± 0.10 ^b^	26.04 ± 3.44 ^b^	2.64 ± 0.41 ^b^	2.72 ± 0.23 ^b^	0.31 ± 0.03 ^b^	1.45 ± 0.12 ^b^
SAA		3.17 ± 0.61 ^c^	17.09 ± 5.80 ^b^	2.18 ± 0.67 ^c^	48.88 ± 12.33 ^b^	0.93 ± 0.20 ^c^	25.82 ± 3.97 ^b^	2.59 ± 0.44 ^c^	2.67 ± 0.26 ^c^	0.27 ± 0.09 ^c^	1.39 ± 0.17 ^c^
AMA		3.38 ± 0.59 ^b^	17.03 ± 4.71 ^b^	2.15 ± 0.81 ^c^	44.46 ± 11.43 ^c^	0.88 ± 0.17 ^d^	24.45 ± 3.84 ^c^	2.46 ± 0.33 ^d^	2.63 ± 0.30 ^d^	0.26 ± 0.08 ^d^	1.35 ± 0.17 ^d^
ASA		3.43 ± 0.38 ^ab^	16.20 ± 5.77 ^c^	1.99 ± 0.37 ^d^	42.79 ± 4.53 ^cd^	0.88 ± 0.05 ^d^	23.88 ± 2.40 ^d^	2.32 ± 0.20 ^e^	2.53 ± 0.18 ^e^	0.24 ± 0.04 ^e^	1.34 ± 0.13 ^e^
CON		3.47 ± 0.70 ^a^	16.02 ± 6.69 ^c^	1.94 ± 0.33 ^d^	42.16 ± 3.81 ^d^	0.87 ± 0.04 ^d^	22.24 ± 4.37 ^e^	2.21 ± 0.44 ^f^	2.50 ± 0.42 ^e^	0.23 ± 0.04 ^e^	1.31 ± 0.26 ^e^

** and ns, significant at 1% level of probability and non-significant, respectively. Pro, proline; TSS, total soluble sugars; CAT, catalase activity; SOD, superoxide dismutase activity; APX, ascorbate peroxidase activity; TPC, total phenol content; TFC, total flavonoid content; N, nitrogen; P, phosphorus; K, potassium.

## Data Availability

Data are available from the corresponding author upon reasonable request.
