# Correction: Abdali et al. The Role of Stress Modifier Biostimulants on Adaptive Strategy of Oregano Plant for Increasing Productivity Under Water Shortage. *Plants* 2023, *12*, 4117

**DOI:** 10.3390/plants15213397

**Published:** 2026-11-02

**Authors:** Reza Abdali, Amir Rahimi, Sina Siavash Moghaddam, Saeid Heydarzadeh, Carmen Arena, Ermenegilda Vitale, Mohammad Zamanian

**Affiliations:** 1 Department of Plant Production and Genetics, Faculty of Agriculture and Natural Resources, Urmia University, Urmia P.O. Box 165-57153, Iran; 2 Department of Plant Production and Genetics, Faculty of Agriculture and Natural Resources, University of Mohaghegh Ardabili, Ardabil P.O. Box 179, Iran; 3 Department of Biology, University of Naples Federico II, 80126 Napoli, Italy; 4 NBFC—National Biodiversity Future Center, 90133 Palermo, Italy; 5 Seed and Plant Improvement Institute, Agricultural Research, Education and Extension Organization (AREEO), Karaj P.O. Box 31585-4114, Iran

**Text Correction**

In the original publication [1], there are errors in the calibration curve standard in the Subparagraph 4.3.5 Pro-line Content, and in the Subparagraph 4.3.6 Total Soluble Sugars (TSS) Determination. Additionally, the measurement units of Total Soluble Sugar (TSS) and Proline Content (Pro) have been corrected. These corrections have been made to the manuscript as detailed below:

**Abstract**

In the abstract, “4.17 μg g^−1^ FW and 27.26 μmol g^−1^ FW” have been replaced with “4.17 mg g^−1^ FW and 27.26 mg g^−1^ FW”.

**2. Results**

2.2.1. TSS and Pro Content

In Section 2.2.1, “4.17 μg g^−1^ FW, 2.35 μg g^−1^ FW, 27.26 μmol g^−1^ FW and 10.28 μmol g^−1^ FW” have been replaced with “4.17 mg g^−1^ FW, 2.35 mg g^−1^ FW, 27.26 mg g^−1^ FW and 10.28 mg g^−1^ FW”.

**Figure 5.**

The measuring units on the Y axis have been corrected accordingly, as shown below.

**Figure 5:**
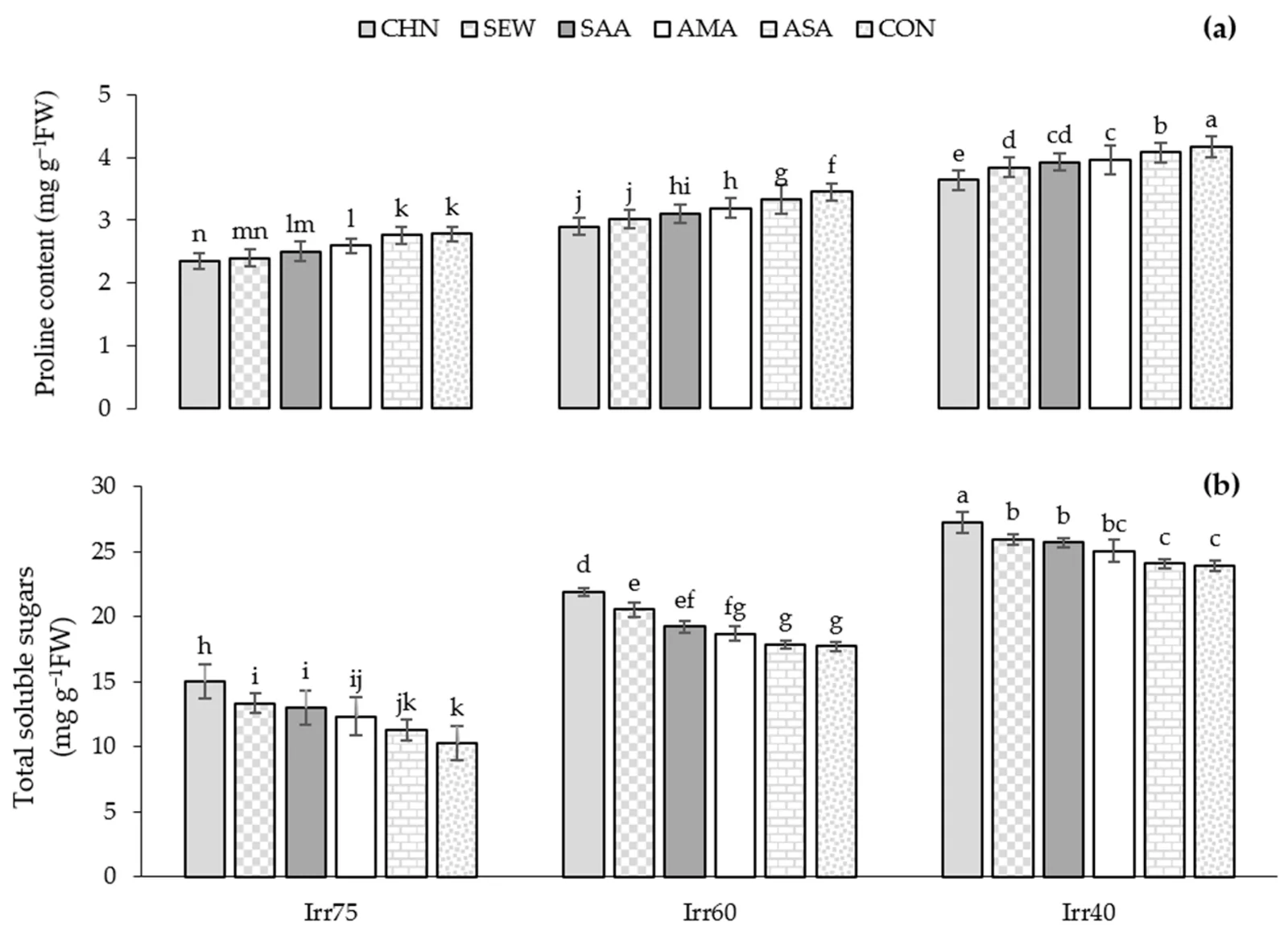
Means comparison and the interactive influence of stress modulators and irrigation regime use on the content of Pro (**a**) and TSS (**b**). Similar letters indicate non-significant differences by LSD test at *p* ≤ 0.05. CHN, chitosan; AMA, amino acids; SEW, seaweed; ASA, ascorbic acid; SAA, salicylic acid; CON, control; Irr40, 40, Irr60, 60, and Irr75, 75% field capacity.

**Table 2.**

In the columns “Pro and TSS”, the measuring units “μg g^−1^ FW and μmol g^−1^ FW” have been replaced with “mg g^−1^ FW and mg g^−1^ FW”, respectively.

**4. Materials and Methods**

The corrections have been made as follows:

4.3.5. Proline Content

Proline content was performed by ninhydrin reagent protocol following the procedure reported in Paquin and Lechasseur [59], using 0.5 g of fresh leaf. The absorbance was evaluated at 515 nm by Spectronic 20 colorimeter (SP 6-200 Unican Montréal-Est, Quebec, QC, Canada). L-proline was used as standard to make a reference curve (R^2^ = 0.99, ranging from 0 to 150 mg L^−1^). Proline content was established from the reference curve and expressed as mg g^−1^ of FW.

4.3.6. Total Soluble Sugars (TSS) Determination

Leaf TSS was performed, as founded by the phenol-sulfuric acid methodology [60]. In this manner, 0.5 g of fresh leaves was homogenized with ethanol. The extract was filtered and treated with 5% phenol and 98% sulfuric acid. This mixture was left for 1 h and its absorption was measured by spectrophotometer at 625 nm. For TSS determination a standard curve (R^2^ = 0.99, ranging from 0 to 80 mg L^−1^) was produced by plotting solutions with varying D-glucose concentrations. The results were assessed through the standard curve and expressed as mg g^−1^ of FW.

The authors state that the scientific conclusions are unaffected. This correction was approved by the Academic Editor. The original publication has also been updated.
